# Hemorrhage and secondary infection of an infantile hemangioma during involution: A case report

**DOI:** 10.1002/ccr3.3989

**Published:** 2021-05-06

**Authors:** James Reed Gardner, Colin Fuller, Adam B. Johnson

**Affiliations:** ^1^ Department of Otolaryngology‐Head and Neck Surgery University of Arkansas for Medical Sciences Little Rock AR USA; ^2^ Division of Pediatric Otolaryngology Arkansas Children's Hospital Little Rock AR USA

**Keywords:** hemorrhage, infantile hemangioma, intramuscular, vascular anomaly

## Abstract

Bleeding complications due to IHs are generally limited. Secondary abscess formation after hemorrhage has not been reported. Rapid expansion of an IH should raise concern for hemorrhage in IHs that have been an involutionary phase.

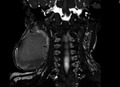

## 1 INTRODUCTION

Infantile hemangiomas (IHs) are the most common tumors of infancy. The vast majority of these tumors spontaneously involute over time. Herein, we report a patient with a pathologically proven intramuscular IH who experienced hemorrhage at age 14 months, complicated by secondary abscess formation that necessitated surgical management.

Infantile hemangiomas (IH) are the most common tumors in infancy, occurring in approximately 5% of infants.^1^ This benign vascular tumor typically proliferates until age 3‐5 months and then gradually regresses spontaneously with involution beginning by 1 year of age.^2^, ^3^ However, 10%‐15% of IHs require treatment due to complications.^3^

We report an unusual case of an intramuscular hemangioma complicated by late‐onset acute hemorrhage and suppurative infection that required surgical management. The diagnosis was ultimately confirmed pathologically.

## 2 CASE REPORT

Our patient initially presented to our otolaryngology clinic at 4 months of age with swelling in the right posterior neck that had undergone outside computed tomography (CT) imaging with intravenous contrast. Imaging was consistent with an intramuscular hemangioma involving the deep aspect of the right sternocleidomastoid muscle. He had already been diagnosed with a small, 1 mm IH of his right leg that was not undergoing treatment. After a normal electrocardiogram was obtained, propranolol therapy was initiated at 2 mg/kg/day. He followed a typical treatment course until 11 months of age when he returned to our clinic with concerns for recent growth after a period of involution. A neck ultrasound demonstrated slight increase in size of the lesion compared with prior CT. An injection of triamcinolone was performed in the clinic, and a 4‐month follow‐up visit was planned. One month later, a propranolol taper was initiated as he had reached 12 months of age.

**FIGURE 1:**
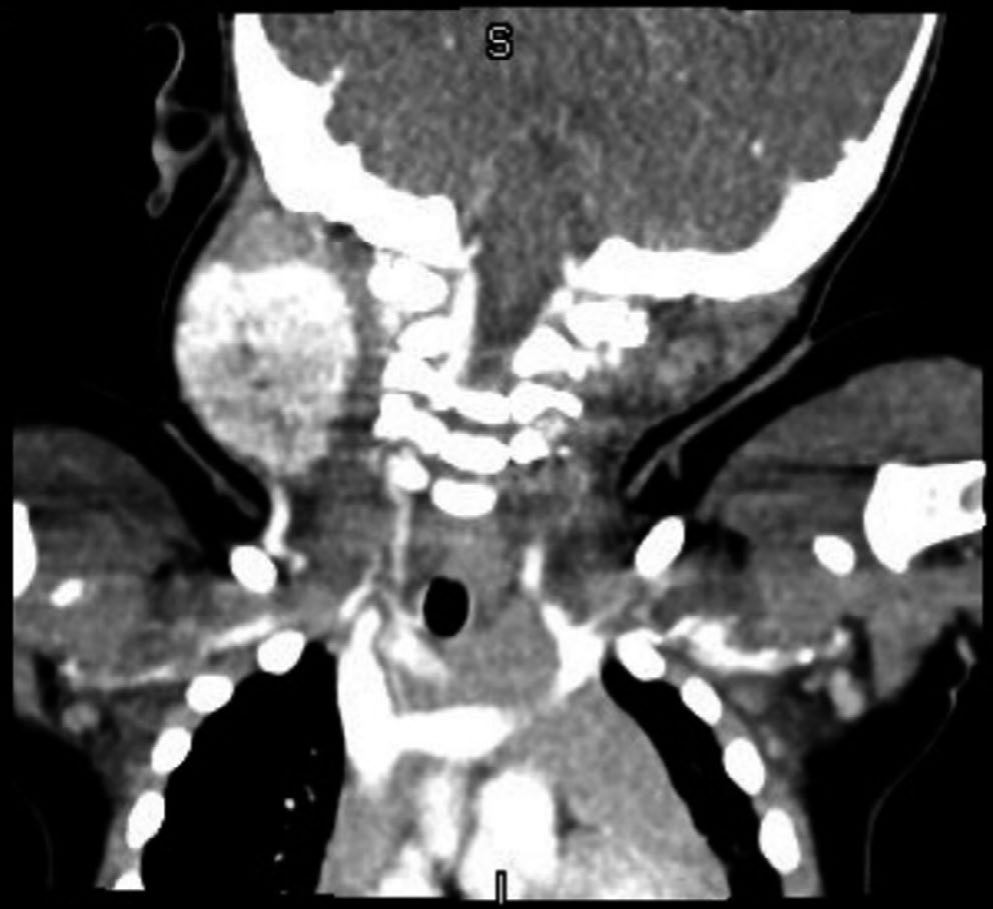
Prior CT demonstrating the size of the IH approximately 1 y prior

**FIGURE 2:**
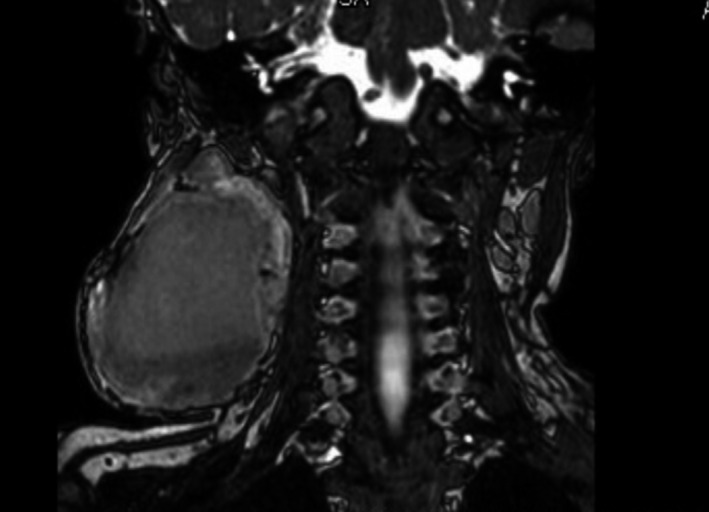
Magnetic resonance imaging indicating central cavitation of IH with peripheral hemosiderin, consistent with intralesion hemorrhage, at age 14 mo

**FIGURE 3:**
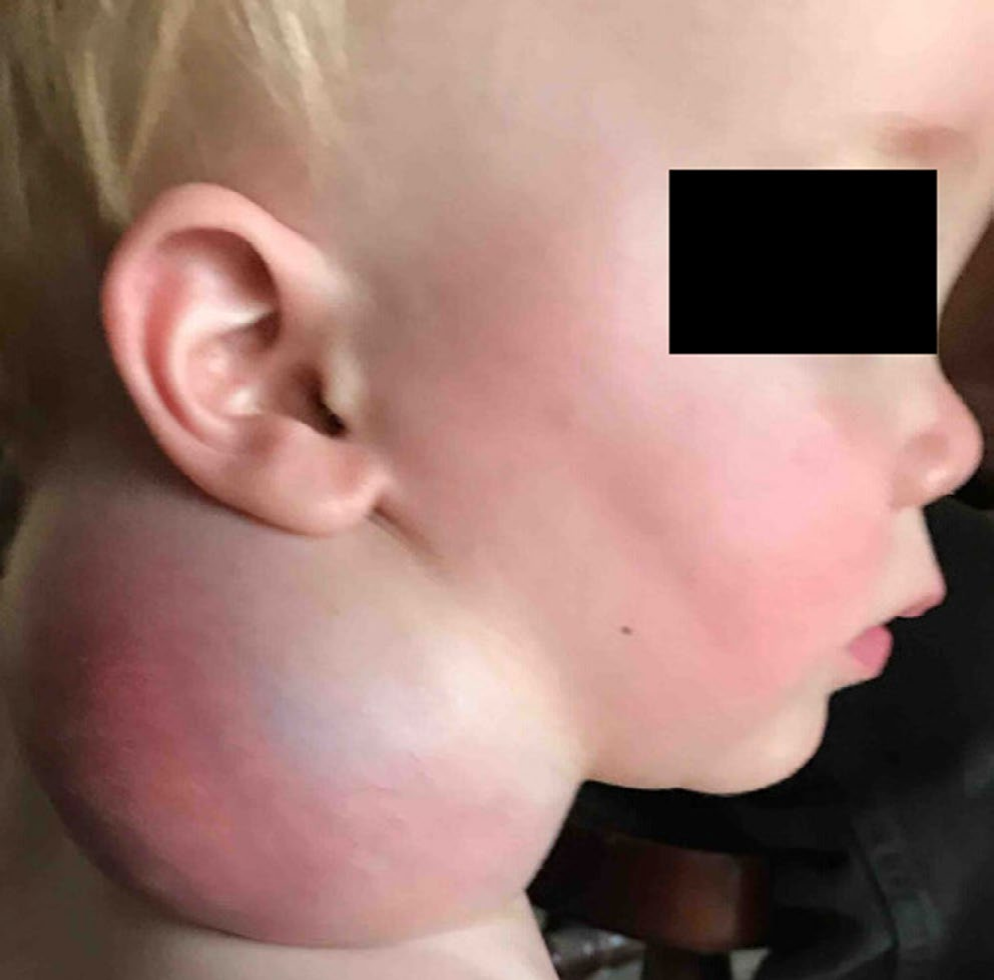
Acutely infected intralesional hematoma

Although improvement occurred, 3 months after the injection he presented to the emergency department (ED) with new expansion of his neck lesion. There was no history of preceding neck trauma. He was subsequently returned to a normal weight‐based dose of propranolol and discharged home. Outpatient magnetic resonance imaging (MRI) showed enlargement of the IH with central cavitation compared with imaging studies 1 year prior (Figures 1,2). The central cavity was noted to be lined by hemosiderin, consistent with hemorrhage. He returned to the ED approximately 4 days later with impressively large acute swelling of his neck accompanied by erythema, pain on palpation, fever, and poor oral intake for approximately 2 days (Figure 3). Hematologic studies showed an elevated CRP and white blood cell count.

Computed tomography (CT) of the neck showed a larger fluid collection in the right posterior neck. Concern for abscess formation was also noted due to the presence of air within the collection. The patient was started on Clindamycin with planned excision the following day.

In the operating room, purulence was quickly encountered upon dissection of the abscess capsule. The associated mass was dissected off of the posterior border of the sternocleidomastoid and internal jugular vein and removed with the abscess capsule en bloc. Cranial nerve XI was identified and preserved during dissection. The wound was closed primarily with a suction drain. Cultures ultimately grew Group A *Streptococci*. The patient had no difficulties with oral intake postoperatively and was discharged on the second postoperative day after removal of the drain. The mass was confirmed by pathology to be an infantile hemangioma via GLUT‐1 immunohistochemistry, with an associated abscess cavity.

## 3 DISCUSSION

This case presents a deep IH with an unusual clinical course and complication. IHs can be classified as focal or segmental. Focal IH can be further subclassified as superficial, deep, or compound.^4^ Superficial IHs are confined to the upper dermis, while deep IHs occupy deeper soft tissues, with or without a superficial component.

Deep IHs usually present at 2‐3 months of age, which notable as it is 1‐2 months later than superficial lesions.^3^ While deep hemangiomas typically follow a similar pattern of growth and involution,^2^ it is possible to observe a prolonged proliferative phase up to 1 year of age and an involution phase of up to 7‐8 years of age.^3^ Of the IHs requiring treatment, 10%‐15% are either nonresponsive or display regrowth after propranolol therapy. Both are more common in deep IHs than superficial IHs.^3^ In these cases, corticosteroids represent a useful therapeutic option. These differences in progression and treatment response were thought to explain the initial expansion of the lesion leading to the adjuvant use of an intralesional triamcinolone injection.

The subsequent presumptive diagnoses of infantile hemangioma hemorrhage and secondary infection, upon representation, were both made via sequential imaging studies. Unfortunately, the diagnosis was delayed several days by the need for patient travel to our tertiary care children's hospital and the family's reluctance to schedule an MRI following the initial ED visit. Hemorrhage in the present case also raises the possibility that the IH was in a prolonged growth phase. While deep IHs are known to have more prolonged growth phases than their superficial counterparts, at age 14 months even an intramuscular IH would be expected to be undergoing involution, which caused our team to question the presumptive diagnosis until confirmed by microbiology and pathology after surgical intervention.

This case demonstrates an extremely rare occurrence of a supposedly involuting IH experiencing a hemorrhagic event. Fortunately, the hemorrhage was contained, and not hemodynamically significant. The resulting secondary infection makes this case even more rare. Most of the reported bleeding complications of IHs correspond with ulceration and minimal blood loss.^5^ To the authors' knowledge, there have been no reports of IH hemorrhage complicated by superimposed abscess formation in a previously stable, involuting IH. Though there is a possibility that the triamcinolone injection may have caused bacterial seeding of the needle tract or injury to local structures, the presentation of an acutely infected abscess 3 months later from one of these processes makes this possibility highly unlikely to have occurred. This report underlines the clinical variation in proliferative and involution phases in deep and superficial IHs.

## 4 CONCLUSIONS

Bleeding complications due to IHs are generally limited. There are few reported cases of large volume hemorrhages related to ulceration of IHs.^5^ Until the present case, secondary abscess formation after hemorrhage has not been reported. Rapid expansion of an IH should raise concern for hemorrhage even in hemangiomas that are expected to be involuting based on the timing of presentation.

## CONFLICT OF INTEREST

The authors report no personal or financial conflicts of interest.

## AUTHOR CONTRIBUTIONS

JRG: contributed to design, data acquisition, and drafting and revision of manuscript. CF: contributed to conception, design, and drafting and revision of the manuscript. ABJ: contributed to the conception, design, and drafting and revision of the manuscript.

## ETHICAL APPROVAL

Approval was obtained from the Institutional Review Board (IRB# 202187).

## Data Availability

The data that support the findings of this study are available on request from the corresponding author. The data are not publicly available due to privacy or ethical restrictions.
